# *In Vitro* Susceptibilities of Wild and Drug Resistant *Leishmania donovani* Amastigote Stages to Andrographolide Nanoparticle: Role of Vitamin E Derivative TPGS for Nanoparticle Efficacy

**DOI:** 10.1371/journal.pone.0081492

**Published:** 2013-12-10

**Authors:** Subhasish Mondal, Partha Roy, Suvadra Das, Asim Halder, Arup Mukherjee, Tanmoy Bera

**Affiliations:** 1 Division of Medicinal Biochemistry, Department of Pharmaceutical Technology, Jadavpur University, Kolkata, West Bengal, India; 2 Department of Chemical Technology, University of Calcutta, Kolkata, West Bengal, India; The Ohio State University, United States of America

## Abstract

Visceral leishmaniasis (VL) is a chronic protozoan infection in humans associated with significant global morbidity and mortality. There is an urgent need to develop drugs and strategy that will improve therapeutic response for effective clinical treatment of drug resistant VL. To address this need, andrographolide (AG) nanoparticles were designed with P-gp efflux inhibitor vitamin E TPGS (D-α-tocopheryl polyethyleneglycol 1000 succinate) for sensitivity against drug resistant *Leishmania* strains. AG loaded PLGA (50∶50) nanoparticles (AGnps) stabilized by vitamin E TPGS were prepared for delivery into macrophage cells infested with sensitive and drug resistant amastigotes of *Leishmania* parasites. Physico-chemical characterization of AGnps by photon correlation spectroscopy exhibited an average particle size of 179.6 nm, polydispersity index of 0.245 and zeta potential of −37.6 mV. Atomic force microscopy and transmission electron microscopy visualization revealed spherical nanoparticles with smooth surfaces. AGnps displayed sustained AG release up to 288 hours as well as minimal particle aggregation and drug loss even after three months study period. Antileishmanial activity as revealed from selectivity index in wild-type strain was found to be significant for AGnp with TPGS in about one-tenth of the dosage of the free AG and one-third of the dosage of the AGnp without TPGS. Similar observations were also found in case of *in vitro* generated drug resistant and field isolated resistant strains of *Leishmania.* Cytotoxicity of AGnp with and without TPGS was significantly less than standard antileishmanial chemotherapeutics like amphotericin B, paromomycin or sodium stibogluconate. Macrophage uptake of AGnps was almost complete within one hour as evident from fluorescent microscopy studies. Thus, based on these observations, it can be concluded that the low-selectivity of AG in *in vitro* generated drug resistant and field isolated resistant strains was improved in case of AG nanomedicines designed with vitamin E TPGS.

## Introduction

*Leishmania donovani* is one of the most common species responsible for visceral leishmaniasis (VL) in India, Bangladesh and Sudan. The pentavalent antimonials are widely used as intramuscular injected treatment of VL, but increase in resistance to this agent has led to investigation of new drugs. The risk of human immunodeficiency virus (HIV) co-infection in patients with VL or kala-azar in endemic areas also has posed a major challenge in control programmes. We undertook this study to identify an alternative to current leishmaniasis treatment. Andrographolide (AG), diterpenoid lactone extracted from the leaves of *Andrographis paniculata,* is a strong antiparasitic and antileishmanial compound [1]. Previous reports revealed antileishmanial, antimalarial [2] and anti-filaricidal [1] activities of andrographolide. Encapsulated andrographolide in liposomes was reported earlier to decrease the spleenic burden of the *L. donovani* parasite in hamster models [3]. An inverse linear relationship was observed between size of the delivery device and antileishmanial activity when an AG derivative, 14-deoxy-11-oxo-andrographolide, was entrapped in various drug carriers [4]. Like most bioactive terpenoids, AG is sparingly soluble in water limiting its biodistribution and localization [2]. Additionally, AG is unstable in extremes of gastrointestinal alkaline and acidic conditions and has a very short biological half life (t_½_ = 2 hours) [5]. Therefore, development of a suitable delivery device for the diterpenes like AG can provide a relatively low cost and indigenous therapeutic lead in neglected tropical diseases like leishmaniasis. D-α-tocopheryl polyethyleneglycol 1000 succinate (vitamin E-TPGS, or simply TPGS) is a water-soluble derivative of natural vitamin E and it is currently approved by USFDA for use as an excipient in various nanoparticle formulations [6], [7]. TPGS has amphiphilic structure having lipophilic alkyl tail and hydrophilic polar head groups with a HLB value of 13.2 and a critical micelle concentration (CMC) of 0.02% w/w [6]. Vitamin E TPGS adds multiple advantages like extended half-life of the drug in plasma, enhancement of drug loading capacity (77 times higher than polyvinyl alcohol) and enhancement of cellular uptake of the drug [8], [9]. Multiple resistance mechanisms have been described in resistant *Leishmania* species. Sb(V), a prodrug, have to be converted to Sb(III) in order to be active. Parasite specific thiol dependent reductase 1 (TDR1) and ACR2 enzymes were characterized in *Leishmania* and was shown to reduce Sb(V) to Sb(III) [10], [11]. Alternatively, there is evidence that a number of thiols, including parasite-specific thiols such as trypanothione as well as macrophage-specific thiols such as glycylcysteine, can reduce Sb(V) to Sb(III) non-enzymatically [12]. Down regulation of TDR1, ACR2 and lower rate of thiol biosynthesis may lead to Sb(V) resistance. Resistant isolates compared to antimony sensitive showed enhanced expression of thiol metabolizing enzymes in varying degrees coupled with increased intracellular non-protein thiol content. Macrophages infected with resistant but not with sensitive strains showed up-regulation of the ATP Binding Cassette (ABC) transporter multidrug resistance protein 1 and permeability glycoprotein [13]. Multidrug resistance (MDR) is a major problem, limiting the treatment of leishmaniasis. Several molecular mechanisms have been proposed in the development of a drug resistant phenotype [14]. One of the most studied resistance mechanisms is the reduction of intracellular drug concentration by efflux proteins that pump drugs out of the cells before they reach the site of action. Many of these efflux proteins are the members of the ATP-binding cassette transmembrane protein super-family, including P-glycoprotein (P-gp) and MDR protein-1 (MRP-1). P-gp was first described and the best characterized to date [15]. P-gp uses ATP as energy to transport drugs and other xenobiotics from the intracellular to the extracellular compartments. The localization of P-gp in normal cells suggests that this transport has the physiological function of detoxification and excretion of xenobiotics [16]. Vitamin E TPGS is reported as a P-gp efflux inhibitor [17] and therefore, AG nanoparticles tuned with TPGS is expected to elicit pharmacological response in resistant leishmanial cells.

In our work, andrographolide-loaded PLGA nanoparticles (AGnps) using vitamin E TPGS was fabricated by solvent extraction or evaporation technique. AGnps were characterized by various state-of-the art techniques such as photon correlation spectroscopy (PCS) for particle size, size distribution and zeta potential measurements. Atomic force microscopy (AFM) was used for nanoparticle surface property measurement and high-performance liquid chromatography (HPLC) was used to measure the drug encapsulation efficiency (EE) and study the *in vitro* drug release kinetics. AGnps were further evaluated against wild-type and drug resistant amastigotes of AG83, and drug resistant GE1 amastigotes.

## Materials and Methods

### 1. Materials

Standard glass wears of Borosil® were used for experimental purposes. A 700 MW sonicator, model Vibra cell VCX 750 (Sonics, USA), homozinizer, model TH 02 (Omni International, USA), a precision balance 0.00001 g, Mettler Toledo AL54 (Mettler, USA), an ultracentrifuge, Himac CS120GHXL (HitachiKoki, Japan) were used in preparative processes. Chemical analyses were carried out by Waters dual pump HPLC model 515 (Waters, USA). Zetasizer Nano ZS (Malvern, UK), Atomic Force Microscope Nanoscope 3A (Veeco, England), FEI Tecnai TM Transmission Electron Microscopy (Netherland), FT-IR 670 Plus (Jasco, Japan) and fluorescence microscope (BX51, Olympus, USA) were used for most particle characterization and imaging experiments. All solvents used were of HPLC grade and of E. Merck or Spectrochem brand. Water used was of milipore grade, Elix 3 Century ZLXS5003Y, (Milipore, USA). PLGA (50∶50, MW 40000–75000), PVA (89,000–98,000), Dialysis tubing D9652 (MW cut off 12,400), FITC (Fluorescein 5(6)-isothiocyanate), D-α-tocopheryl polyethylene glycol 1000 succinate (TPGS, C_33_O_5_H_54_ (CH_2_CH_2_O)_23_), amphotericin B, paromomycin (PMM), medium 199, RPMI-1640 and fetal calf serum were purchased from Sigma Aldrich (USA). Andrographolide was purchased from Natural Remedies (Bangalore, India), while sodium stibogluconate (SSG) was a generous gift from Albert David Ltd. (Kolkata, India). GraphPad Prism 5.01 and Sigmaplot 6.0, (Zendal Scientific, USA) were used for the data analysis purposes.

### 2. Ethics Statements

BALB/c mice of either sex, weighing 20–25 g and of approximately the same age were used for the study. The experimental protocols were approved by the Jadavpur University Animal Ethics Committee, and procedures followed were in accordance with the Committee’s guidelines, with necessary humane care. Mice were housed in polypropylene cages and fed with a standard diet and water ad libitum. Mice were exposed to a normal day and night cycle.

### 3. Preparation of Andrographolide Nanoparticles (AGnps)

AG nanoparticles were prepared by following an emulsion solvent evaporation technique. Briefly, 50 mg PLGA and 2.5 mg of AG were dissolved in 3 ml of chloroform. This phase was emulsified by sonication for 1 minute in 0.04% (w/v) of aqueous TPGS solution at 20 KHz. Resultant O/W emulsion was homogenized for 20 minutes at 20,000 r.p.m. under external cooling in ice water. The solvent evaporation was continued for 12 hours over a magnetic stirrer at room temperature. The nanoparticles (AGnps) formed were harvested by ultracentrifugation at 30,000 r.p.m. for 25 minutes at 4°C. AGnps were washed with water, recentrifuged and preserved in vacuum desiccators at 4°C until further evaluations. AG nanoparticles without TPGS were prepared by following the similar technique using macromolecular stabilizer PVA [18]. Fluorescent PLGA nanoparticles (FAGnp) were prepared in a similar fashion where flurophore FITC in same quantity replaced AG.

### 4. Particle Size and Polydispersity Measurements

The particle size of AGnp preparations was measured in Photon Correlation Spectroscopy (PCS), (Zetasizer nano ZS, Malvern Instrument Limited, UK) with a 4 mw He–Ne laser beam of wavelength 633 nm and at a back scattering angle of 173° using NIBS technology.

### 5. Zeta Potential Measurement

Zeta potential of the AGnp preparations was determined using zeta potential PCS cuvettes in Zetasizer nano ZS (Zetasizer nano ZS, Malvern Instrument Limited, UK) based on electrophoretic mobility under an applied electrical field.

### 6. Atomic Force Microscopy and Transmission Electron Microscopy

AGnp suspensions (100 µl) in water were deposited onto fused mica substrates by dropcast method. The particles were visualized in AFM (Veeco Nanoscope IIIa, England) in tapping mode using RTESP tip with 267–328 kHz resonance frequency at a scan speed of 1.2 Hz. The morphology of nanoparticles was examined by the conventional negative staining method with FEI Tecnai TM Transmission Electron Microscopy (Netherland) which was operated with an acceleration voltage of 80 kV. One drop of nanoparticle was placed over the carbon-coated copper grids and then these grids were washed with distilled water. Then staining was performed with uranil acetate solution. Sample was air-dried before observation.

### 7. AG Encapsulation Efficiency Study

Estimation of AG in all cases was carried out by a reverse phase HPLC system. The mobile phase was acetonitrile (0.1% v/v) and phosphoric acid in water (40∶60 v/v) at a flow rate of 1 ml/min. The analysis was carried out by using 250×4.6 mm C_18_ column (Supelco, USA) and a PDA detector (2996, Waters, USA). A peak area (y) vs concentration (x) graph for AG was first prepared, y = 30145x + 68911, R^2^ = 0.9930, retention time was 4.5 min. This was used to detect AG concentrations throughout. Mass of AG in solution before and after nanoparticulation in supernatant was determined by HPLC experiments for calculation of entrapment efficiencies [18].
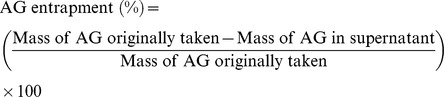



### 8. *In vitro* AG Release Study

AGnp equivalent to 2 mg of AG load was dispersed in 1 ml of phosphate buffer (100 mM, pH 7.4) and the dispersion was carefully transferred into a dialysis bag. The dialysis bags were placed in glass vials containing 10 ml of phosphate buffer and maintained at 37°C in a shaker bath. At predetermined time intervals, the release medium in vials was taken out and replaced with fresh buffer medium. The amount of AG released for timed sample was estimated by HPLC. Standard curve plot was used for analysis with necessary corrections for the dilution factors. The in vitro release and kinetics of the AGnp preparation were studied in the same way. In order to understand the drug release mechanism, the release data was fitted to Higuichi and Korsemeyer-Peppas model. The release exponent n and K value were calculated using sigma plot 6.0 software.

### 9. Storage Stability Study

Triplicate samples of nanoparticles were sealed in amber colored glass vials under nitrogen atmosphere and were stored in the refrigerator (4±0.5°C) for three months. Aliquots were withdrawn at the end of the 1st, 2nd and 3rd month, dispersed in phosphate buffer (100 mM, pH 7.4) for the determination of particle size, polydispersity and zeta potential. AG entrapment in nanoparticles at the end of each month was analyzed by HPLC [19].

### 10. Assay of Nanoparticle Cellular Uptake

In order to study nanoparticles uptake in macrophage cells, the desired cell concentration (4×10^5^ cells/well) was seeded with FAGnp (0.5 mg/ml) and incubated in CO_2_ incubator at 37°C for 15, 30 and 60 minutes time intervals. Samples were withdrawn after these time intervals and observed in FITC channel under fluorescence microscope.

### 11. Parasites and Culture Conditions

Promastigotes of *Leishmania donovani* clones, AG83 (MHOM/IN/83/AG83) and GE1 (MHOM/IN/80/GE1F8R) were VL isolates obtained as a gift from Indian Institute of Chemical Biology, Council of Scientific and Industrial Research, Kolkata, India. Antimony-sensitive strain, AG83 and antimony-resistant isolate, GE1 were characterized earlier [20]–[24]. AG83 is used to consider as reference standard strain of *L. donovani* in India. Parasites were routinely grown as promastigotes in medium 199 supplemented with 10% heat–inactivated fetal calf serum (FCS) at 24°C.

### 12. Resistance Selection to Sodium Stibogluconate and Paromomycin on Promastigotes and their Transformation into Drug Resistant Amastigotes

The wild-type AG83 and resistant field isolate GE1 promastigote cells were cultured in medium 199 (with supplements), in the presence of drug concentration corresponding to the 50% inhibitory concentration (IC_50_) of the strain. The cultures were stabilized for three subcultures before increasing the drug concentration. Drug concentration was increased in such a way that the cell population was decreased approximately 20% for each batch. Finally when 90% cell population of the initial count was reduced, the phenotype so generated was plated on medium 199 agar plates in the presence of same drug concentration, and a single colony was picked for culture in medium 199 liquid media at the same drug concentration [25], [26]. Sodium stibogluconate and paromomycin resistant phenotype clones were designated as AG83-R. Stability of resistance was checked at four, eight and sixteen weeks after removal from drug pressure. Preliminary evidence for the generation of drug resistant *Leishmania donovani* cells had already been published [27].

### 13. Generation of Axenic Amastigotes

*Leishmania donovani* amastigote forms were grown and maintained as described by Debrabant *et al.* [28]. Axenically grown amastigotes of *L. donovani* were maintained at 37°C in 5% CO2/air by weekly sub-passages in MMA/20 at pH 5.5 in petri dishes [29]. Under these conditions, promastigotes differentiated to amastigotes within 120 hours. Cultures were maintained by 1∶3 dilutions once in a week.

### 14. Cross–resistance and Sensitivity Studies

Cross-resistances of sodium stibogluconate (SSG) resistant AG83 to paromomycin (PMM), and paromomycin resistant AG83 to sodium stibogluconate were determined by measuring the 50% inhibitory concentration (IC_50_) by cell counting method using hemocytometer. Similarly, sensitivity (IC_50_) of SSG to SSG resistant AG83, sensitivity of PMM to PMM resistant AG83 and IC_50_s of SSG and PMM to GE1 strain were determined by cell counting. To measure the IC_50_ values, the parasites were seeded into 96-well plates at a density of 1×10^4^ promastigotes/well or 1×10^5^ amastigotes/well in 200 µl medium containing 10 µl of different drugs. Parasite multiplications in treated plates were compared to that of untreated control (100% growth). After 72 hours of incubation, cell count was taken microscopically. The results were expressed as the percentage of reduction in the parasite number compared to that in untreated control wells, and the IC_50_ value was calculated by linear regression analysis (MINITAB V.13.1, PA) or linear interpolation [30].

### 15. Amastigotes in Macrophage Drug Susceptibility Assay

BALB/c mice were injected intraperitonealy with 1.5 ml of 3% thioglycolate medium. After 96 hours, the peritoneal macrophages were harvested by peritoneal lavage using cold RPMI-1640 medium. Cells were counted, centrifuged and re-suspended at a concentration of 4×10^5^/ml in RPMI-1640 medium without supplements. Sterile round glass cover slips (12 mm) were placed in each well of 24-well culture plates. Macrophages were pipetted at a volume of 500 µl/well and allowed to attach for 2 hours at 37°C in 5% CO_2_. After 2 hours, the medium was removed from the wells and replaced with 500 µl of warm (37°C) RPMI-1640 medium supplemented with 10% FCS, 20 mM L-glutamate, 16 mM NaHCO3, penicillin (50 U/ml) and streptomycin (50 µg/ml). At the following day, a suspension of 4×10^6^ amastigotes in RPMI-1640 was added in a 500 µl volume to each well (for a macrophage/parasite ratio of 1∶10). The plates were incubated for 4 hours at 37°C in 5% CO_2_ and the medium was aspirated to remove free parasites. Fresh 1 ml RPMI-1640 with or without drugs or nanoparticles at the appropriate concentration was added in triplicate wells. Plates were incubated for 72 hours at 37°C in 5% CO_2_. The medium was aspirated and the cover slips were removed and then methanol fixed and air dried. After staining with Giemsa, 100 cells on the glass disks were counted. Three independent experiments in triplicate for each concentration were performed for efficacy of drugs or nanoparticles. Results were presented as the ratio between the infection proportions of treated and untreated macrophage cultures.

### 16. Cytotoxicity Assay and Selectivity Index

Macrophages cells were cultured in RPMI-1640 medium supplemented with 10% FCS, 20 mM L-glutamate, 16 mM NaHCO3, penicillin (50 U/ml) and streptomycin (50 µg/ml). The assay was performed in 96-well tissue culture plates in the presence of standard counts of macrophages. The wells were seeded with test solutions and the viable macrophages were counted microscopically.

A key part of drug discovery and development is the characterization and optimization of the safety and efficacy of drug candidates and to identify those that have an appropriately balanced safety efficacy profile for a given indication. The selectivity index (SI) which is typically considered as the highest exposure to the drug that results in no toxicity to the exposure that produces the desired efficacy – is an important parameter in efforts to achieve this balance. In the present study, the degree of selectivity of the drug or its formulation is expressed as SI = CC_50_ of a drug or its formulation in a macrophage cell line/IC_50_ of the same drug or its formulation, where CC_50_ is the concentration required to kill 50% of the host cell population and IC_50_ is the concentration required to kill 50% of the parasites inside the host cell. When the SI value is ≥10, that drug or formulation presents promising activity, that is higher than its cytotoxicity [31].

### 17. Statistical Analysis

Experimental results were expressed as mean ± standard deviation. Student’s t test was used to calculate the statistical difference of mean values. Differences were considered significant at a level when p<0.01.

## Results

Limited solubility window of AG was a major constraint in the preformulation stages of nanoparticle preparation. Chloroform was used as a solvent for both AG and PLGA during optimization experiments in particle size and drug loading experiments. Nanoparticles were prepared by emulsion solvent evaporation technique using both sonication and homogenization. These two processes when combined produced gaussian size distribution and relatively small particle size than other experiments, where only homogenization step was used [32]. PLGA nanoparticle preparations in a sequential application of sonication and homogenization provided a better control for particle sizing as well as drug entrapment. In our work, vitamin E TPGS was chosen as a stabilizer over PVA as used earlier [18] because of some specific advantages. Vitamin E TPGS has a hydrophilic domain comprising of PEG and a lipophilic domain of tocopherol succinate, thereby making it plausible for use as a surface active agent. Ease of removal of residual TPGS from the nanoparticle surface by washing had given it a definite edge over PVA stabilized nanoparticles where residual polyvinyl pyrrolidone affected the purification of the final product [33]. Furthermore, TPGS behaves as a component of the matrix material and enhances the control release property of the nanoparticles when combined with hydrophobic polymer PLGA [34]. The sole aim of this work was to prepare functional AG nanoparticles to be active against resistant *Leishmania* strains and to apply vitamin E TPGS as P-gp efflux inhibitor for the present purpose.

### 1. Characterization of AGnps and Drug Encapsulation Efficiency

**Figure 1:**
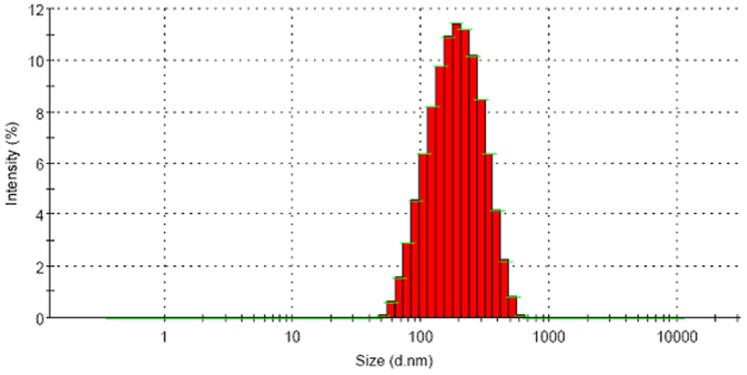
Particle size distribution of andrographolide TPGS nanoparticles. AGnp formulation showed a near perfect Gaussian size distribution. The particle size of AGnp preparations were measured in Photon Correlation Spectroscopy.

The physico-chemical characteristics of the formulated nanoparticles such as particle size and size distribution, zeta potential and drug encapsulation efficiency were summarized in Table 1. The nanoparticles exhibited average particle size of 179.6 nm and polydispersity index (PDI) of 0.245. Size and size distribution of the drug-loaded nanoparticles are crucial parameters in the design of the particulate drug delivery system since they are main factors in determining the drug release kinetics and cellular uptake efficiency of the nanoparticles [35]. AGnp formulation showed a near perfect Gaussian size distribution (Figure 1). Zeta potential of the formulated AGnps was found to be strongly influenced by the emulsifier used in preparation of the nanoparticles. TPGS emulsified nanoparticles exhibited negative surface charge (−37.60 mV) indicating more stable dispersion of nanoparticles. Drug encapsulation efficiency plays a key role in nanoparticle formulations and is directly related to the pharmacodynamic response of the drug. The encapsulation efficiency of AG in PLGA TPGS nanoparticles was found to be 83%.

**Table 1:** Characterization of andrographolide nanoparticle preparations.

Particle size (nm)	Zeta Potential (mV)	Polydispersity Index	AG Entrapment Efficiency (%)
179.6±12.6	−37.60±1.4	0.245±0.01	83.4±3.5

± SD (n = 4). Results expressed as mean

### 2. AFM Surface Topography and TEM Micrograph

**Figure 2:**
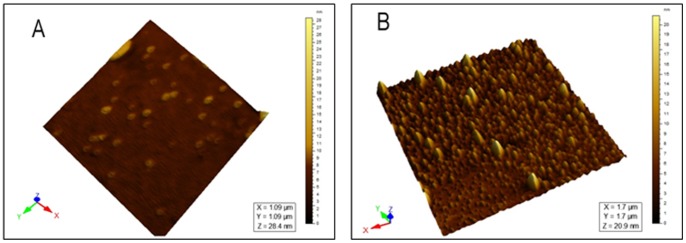
AFM study of AGnps. (A), 2D surface topography; (B), 3D particle distribution. The images revealed that prepared nanoparticles had regular spherical shapes with smooth surfaces in the zone of observation and equipment resolution. No apparent aggregation of nanoparticles was detected.

**Figure 3:**
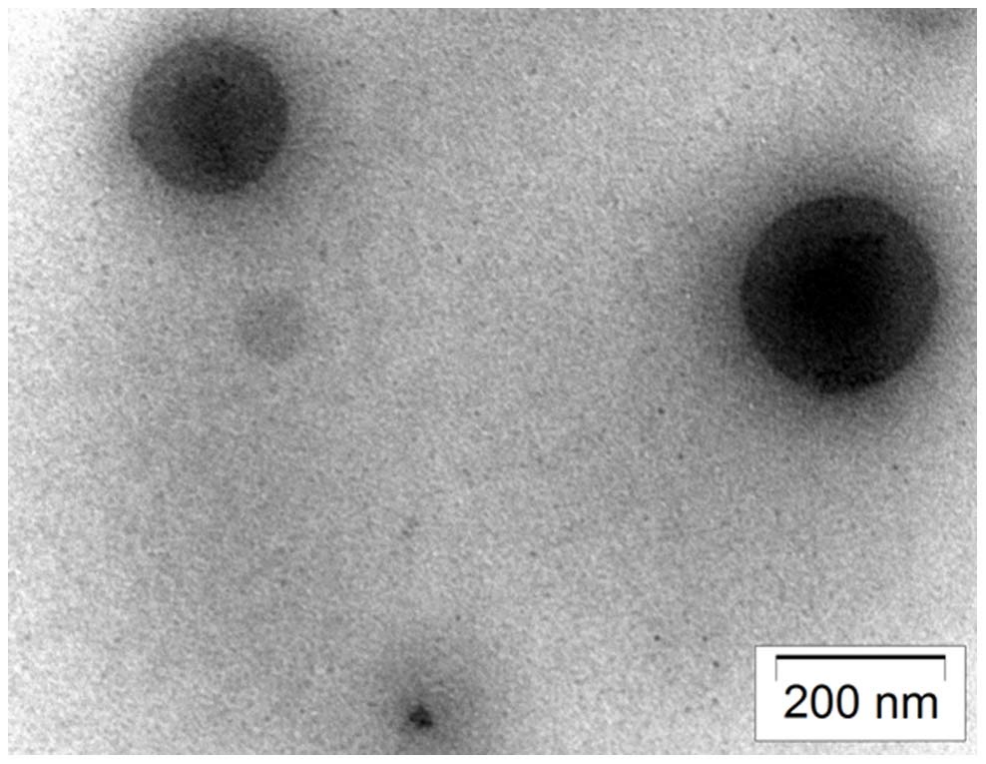
TEM micrograph of AGnps with TPGS obtained from the emulsion solvent evaporation method. One drop of nanoparticle was placed over the carbon-coated copper grids. Sample loaded copper grids were washed with distilled water. Then staining was performed with uranil acetate solution and sample was air-dried before observation. Magnifications: 30,000 X and acceleration voltage: 80 kV.

AFM was extensively used to understand the particle surface morphology of AGnps (Figure 2). The images revealed that prepared nanoparticles had regular spherical shape with smooth surface in the zone of observation and equipment resolution. No apparent aggregation of nanoparticles was detected. Figure 3 showed the TEM image of AGnps with TPGS. It was observed that the particle had a spherical morphology with size smaller than 200 nm.

### 3. Characterization of *in vitro* Drug Release and Release Kinetics

**Figure 4:**
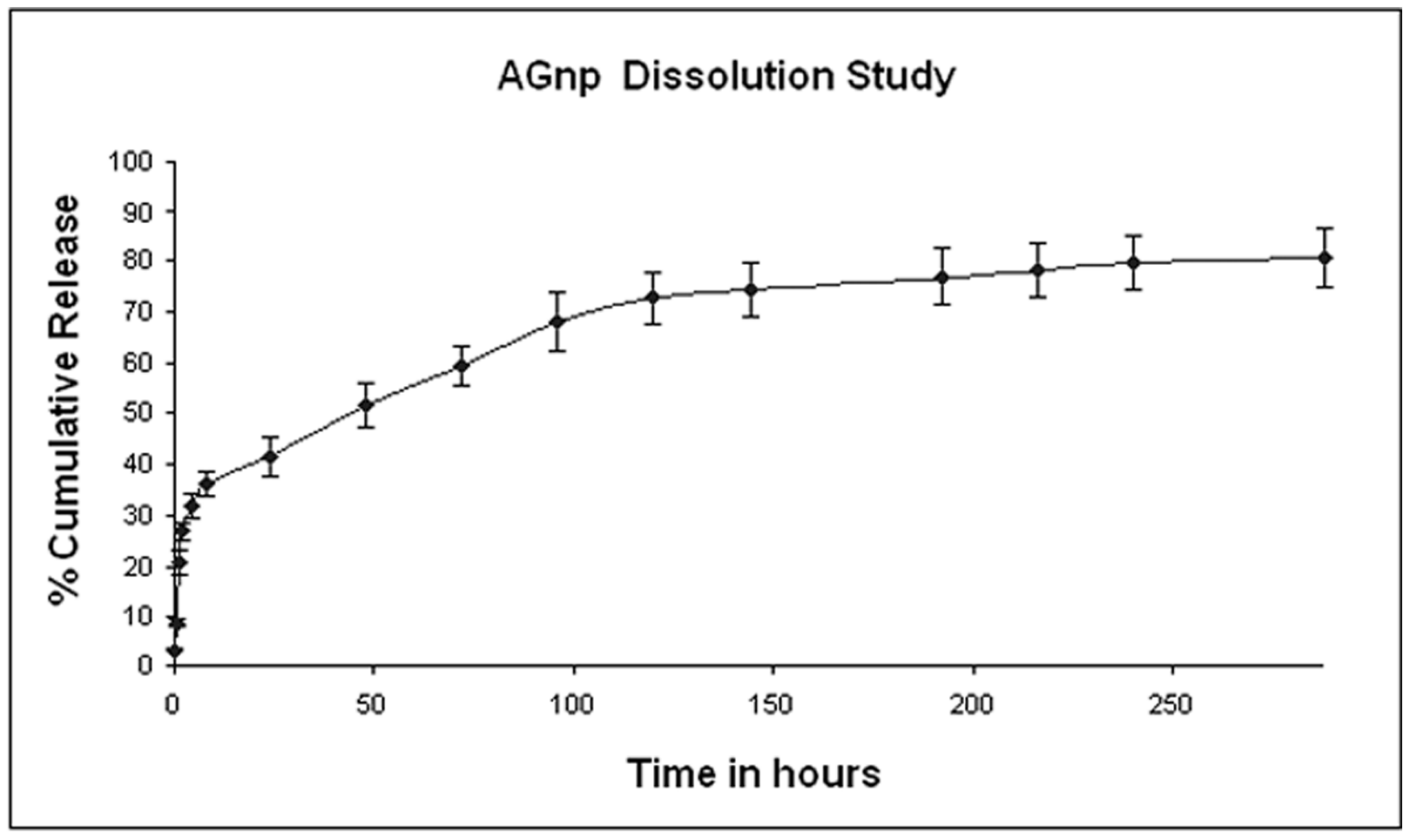
*In vitro* release profile of AGnp preparation. Results expressed as mean ± S.D. (n = 3). The time dependent *in vitro* release of AG was studied and the average percentage cumulative release over time was plotted. The release responses for AGnp preparation was biphasic in nature. A rapid release of AG occurred up to 96 hours followed by a sustained release over a period of 288 hours. The release however, was steady in later hours which continued for a prolonged period of 288 hours.

The time dependent *in vitro* release of AG was studied and the average percentage cumulative release over time was plotted in Figure 4. The release response for AGnp preparation was biphasic in nature. A rapid release of AG occurred up to 96 hours followed by a sustained release over a period of 288 hours. The initial burst release was indicative of some nanoparticle surface adsorbed AG molecules. The release however, was steady in later hours which continued for a prolonged period of 288 hours.

Korsemeyer-Peppas (Equation I) [36] and Higuichi model was used to understand the release mechanism of nanoparticulate delivery devices.
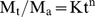
(1)Where, M_t_, mass released at time point ‘t’; M_α_, total mass load; n, the release exponent and K is the constant accommodating the structural and geometric features.

**Figure 5:**
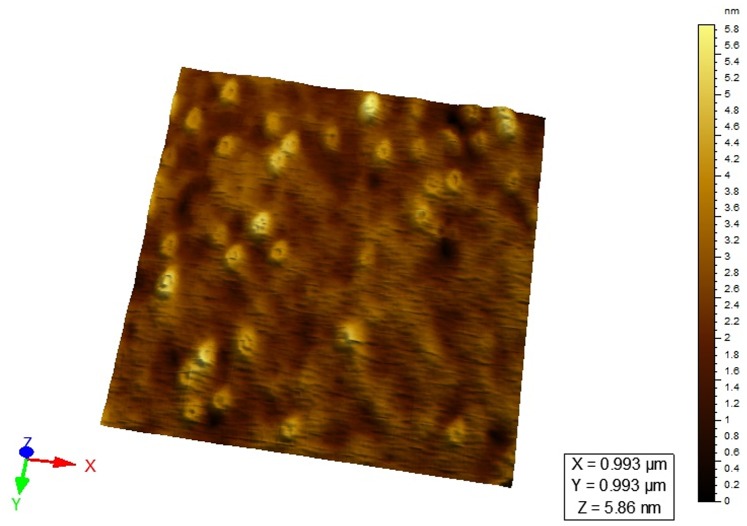
AFM study of AGnps after 192 hours of AG release. AFM studies were carried out separately on particles sampled after 192 hours of dissolution. AGnps sampled after 600 hours of release showed a distinct itching on polymeric nanoparticle surface. This fact clearly indicated that, in later part of dissolution study, nanoparticle surface erosion associated with *in vitro* drug release took place.

The drug release data when fitted into Higuichi model produced a R^2^ value of 0.9236 which indicated improper fit or the release profile was not purely diffusion controlled. This hypothesis was further confirmed by fitting release data into Korsemeyer-Peppas model. The value of release exponents recorded was n = 0.35 and K = 0.137 indicating that the release mechanism was not a simple Fickian diffusion [37] through the polymer matrix. This happens when mass release associated with slow polymer surface erosion or a bulk mass penetration takes place. AFM studies were carried out separately on particles sampled after 192 hours of dissolution. AGnps sampled after 600 hours of release showed a distinct itching on polymeric nanoparticle surface (Figure 5). This fact clearly indicated that nanoparticle surface erosion associated with *in vitro* drug release took place in the later part of dissolution study.

### 4. Nanoparticle Stability Profile

**Figure 6:**
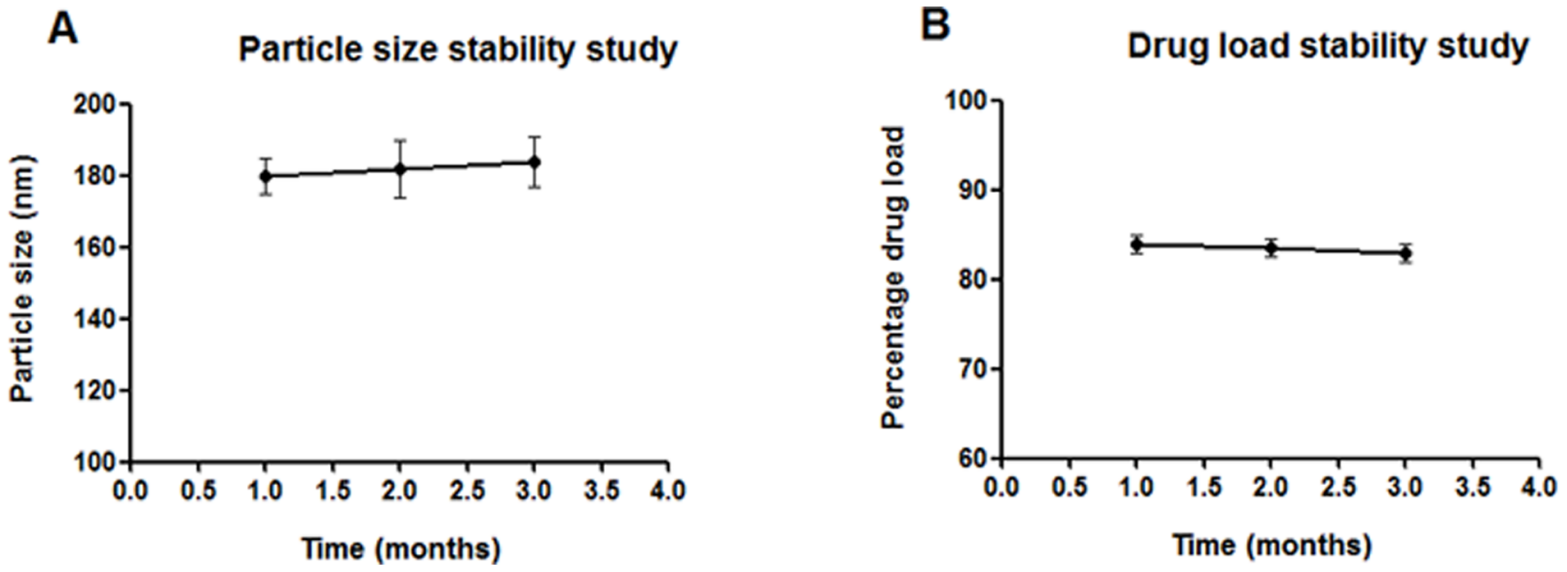
Stability study of andrographolide nanoparticles. (A), Particle size stability study. (B), Drug load stability study. Stability data for AG nanoparticles designed with TPGS stored under refrigeration (4±0.5°C) for a period of three months. During this period AGnps were stable. However, minute AG leaching (nearly 1%) from the nanoparticle preparation was observed after three months.

Stability data for AG nanoparticles designed with TPGS, stored under refrigeration for three months period were represented in Figure 6. TPGS has 77 times higher emulsification efficiency compared to PVA [6] which prevented particle coalescence during the study period. However, minute AG leaching (nearly 1%) from the nanoparticle preparation was observed after three months. This factor might have resulted in slight positive shift of zeta potential from −37.6 mV to −36.2 mV for AG nanoparticles. Similar shift in zeta potential was also observed in case of pluronic stabilized AG nanoparticles reported earlier by our group [19].

### 5. Cellular Uptake of AGnps

**Figure 7:**
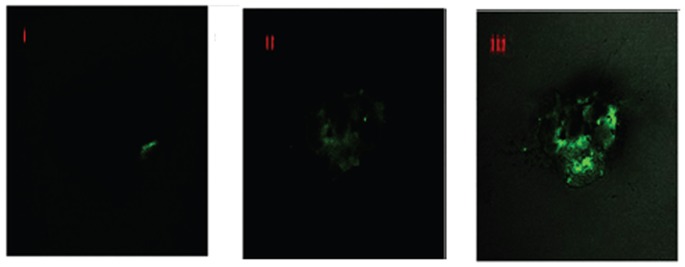
Andrographolide nanoparticles uptake in macrophage cells. Fluorescence image. FITC-loaded PLGA nanoparticles (FAGnps) were used to study the cellular uptake of AGnps in peritoneal macrophage cells as the amastigote cells reside within the phagolysosomes of macrophages. 4×10^5^ macrophage cells/well in three bathes were seeded with FAGnps (0.5 mg/ml) and incubated in CO_2_ incubator at 37°C, for (I), 15 minutes; (II), 30 minutes; and (III), 60 minutes. Samples were withdrawn after these time intervals and observed in FITC channel under fluorescence microscope. The prepared nanoparticles showed considerable cellular internalization within 1 hour of incubation and insignificant internalization within 15 minutes period. Cellular internalization of FAGnps only took place in living cells. This figure represented time dependent uptake of FAGnps.

A separate set of experiments with FITC-loaded PLGA nanoparticles was used to study the cellular uptake of AGnps in peritoneal macrophage cells. The prepared nanoparticles showed considerable cellular internalization within 1 hour of incubation (Figure 7), and insignificant internalization within 15 minutes period. Cellular internalization of FAGnps only took place in living cells. The figure represented time dependent uptake of FAGnps.

### 6. SSG and PMM Resistance Selections on Promastigotes

**Figure 8:**
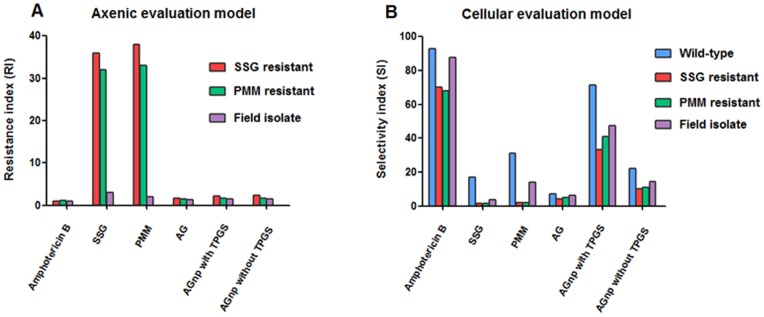
Characterization of resistance index (RI) in axenic *L. donovani* amastigotes and selectivity index (SI) in cellular *L. donovani* amastigotes. Panel A : Nature of resistance indexes (RIs) of amphotericin B, SSG, PMM, AG, AGnp with TPGS and AGnp without TPGS in axenic amastigites of SSG resistant, PMM resistant and field isolate *L. donovani.* RI is defined as given in Table 2. Panel B : Nature of selectivity indexes (SIs) of amphotericin B, SSG, PMM, AG, AGnp with TPGS and AGnp without TPGS in cellular amastigotes of wild-type, SSG resistant, PMM resistant and field isolate *L. donovani.* SI is defined as CC_50_/IC_50_.

Selection process by stepwise drug pressure increase for AG83-R phenotype was found to be limiting at 34 µg Sb(V)/ml for SSG and 165 µM for PMM when 90% of total cell population subjected to death was observed in comparison to untreated cells. When growth rates of finally selected cell lines proved to be fully comparable to that of the parent non-selected strains, promastigotes were transformed to amastigotes and comparison of the IC_50_ values between the parent and the resistance-selected phenotypes revealed large differences, being about 32 to 36 fold SSG resistance for AG83-R. Similarly, AG83-R showed 33 to 38 fold PMM resistance. Field isolate GE1 showed three-fold SSG resistance and two-fold PMM resistance (Table 2, Figure 8). Strikingly, AG showed very low and identical resistance index (RI) values in wild-type and resistant cell lines. When AG was encapsulated in nanoparticles, RI values were remained very low, irrespective of cell types (wild-type or resistant).

**Table 2:** Drug sensitivity profile against *Leishmania donovani* wild-type, drug resistant and field isolated axenic amastigote cell lines.

Drug	IC_50_ (mean ± SD, n = 4) µM^a^						
	Axenic AG83 evaluation model					Axenic GE1 evaluation model	
	Wild-type	SSG resistant^b^	RI^c^	PMM resistant^b^	RI^c^	Field isolate	RI^d^
Amphotericin B	0.2±0.05^f^	0.21±0.05^f^	1.0	0.23±0.05^f^	1.1	0.2±0.05^f^	1
SSG(SbV)^e^	3.6±0.40	130±20	36	115±16	32	11±1.8	3
PMM	10±2^f^	380±40^f^	38	330±30^f^	33	23±3.6^g^	2
AG	160±25^f^	265±38^f^	1.7	225±30^f^	1.4	200±21^f^	1.3
AGnp with TPGS	36±4^f^	75±8^g^	2.1	56±5^f^	1.6	50±7^f^	1.4
AGnp without TPGS	64±8^f^	157±16^g^	2.4	112±10^h^	1.7	90±10^f^	1.4

^a^ Assays are described in Materials and Methods.

*in vitro* as given in Materials and Methods.^b^ SSG and PMM resistant strains were generated

_50_ of AG83 phenotype generated at maximum drug pressure/IC_50_ of wild-type.^c^ RI, Resistance Index was IC

_50_ of field isolate/IC_50_ of wild-type.^d^ RI, Resistance Index was IC

µg Sb/ml.^e^ Values for antimonial agents are in

^f^ p<0.001, significant difference compared with SSG.

^g^ p<0.5, no significant difference compared with SSG.

^h^ p>0.5, no significant difference compared with SSG.

### 7. *In vitro* Susceptibilities of AG Nanoparticles on AG83, AG83–R and GE1 Cellular Amastigotes

It appeared from Table 2 and Table 3 that IC_50_ values for amphotericin B on AG83, AG83-R and GE1 axenic and cellular amastigotes did not differ substantially and consequently resistance index (RI) and selectivity index (SI) remained same for both wild-type and drug resistant strains. It appeared from Table 3 and Figure 8 that the highest SI was found for amphotericin B, whether it was wild-type or resistant cell lines. SI of SSG in wild-type was found to be 17, whereas in resistant SSG, PMM and field isolate strains, SI values were 1.5, 1.6 and 3.5, respectively. Similarly, SI of PMM in wild-type was found to be 31, whereas in resistant SSG, PMM and field isolate strains, SI values were 2, 2.1 and 13.8, respectively. In contrast, similar low SI values of AG were observed in wild-type and resistant strains. Since, these values were less than 10, we might expect that AG itself would not be therapeutically safe. Strikingly enough, when AG was incorporated in nanoparticles, especially with TPGS, SI values were increased massively for wild-type and resistant cell lines.

**Table 3:** Drug sensitivity profile against *Leishmania donovani* wild-type, drug resistant and field isolated intracellular amastigote cell lines.

Drug	IC_50_ (mean ± SD for atleast 4 replicates) µM^a^											
	Cellular AG83 evaluation model								Cellular GE1 evaluation model			
	Wild-type	SI^f^	SSGresistant^b^	RI^c^	SI^f^	PMMresistant^b^	RI^c^	SI^f^	Fieldisolate	RI^d^	SI^f^	CytotoxicityCC_50_ (µM)(Macrophagecells)
Amphotericin B	0.15±0.05^g^	93	0.2±0.05^g^	1.3	70	0.20±0.05^g^	1.3	68	0.16±0.05^g^	1.06	87.5	14±2.1^h^
SSG(SbV)^e^	1.6±0.20	17	18.1±3^g^	11.2	1.5	17.3±2.60	10.8	1.6	7.7±0.65	4.8	3.5	27±3.9
PMM	8±2^g^	31	125±15^g^	15.6	2	115±13^g^	14.4	2.1	18±3^g^	2.2	13.8	248±32^g^
AG	141±20^g^	7.1	230±25^g^	1.6	4.3	200±30^g^	1.4	5	165±27^g^	1.2	6.1	1000±160^g^
AGnp with TPGS	28±2^g^	71.4	60±9^g^	2.1	33.3	49±6^g^	1.8	40.8	42±5^g^	1.5	47.6	2000±300^g^
AGnp without TPGS	54±5^g^	22.2	120±10^g^	2.2	10	107±8^g^	2	11.2	83±9^g^	1.5	14.4	1200±220^g^

^a^ Assays are described in Materials and Methods.

*in vitro* as given in Materials and Methods.^b^ SSG and PMM resistant strains were generated

_50_ of AG83 phenotype generated at maximum drug pressure/IC_50_ of wild-type.^c^ RI, Resistance Index was IC

_50_ of field isolate/IC_50_ of wild-type.^d^ RI, Resistance Index was IC

µg Sb/ml.^e^ Values for antimonial agents are in

_50_/IC_50_.^f^ SI, Selectivity Index was CC

^g^ p<0.001, significant difference compared with SSG.

^h^ p<0.5, no significant difference compared with SSG.

## Discussion

Circumventing the deficiencies of first-line antileshmanial agents has not only scientific importance but also clinical significance. Solubility of the compound is an important factor for the chemotherapeutic agent to be effective. About half of the potentially valuable drug candidates identified by high throughput screening technology (including those with the highest activities) demonstrate poor aqueous solubility. For this reason, the drugs are not developed further, and fail to enter formulation development stage. Andrographolide is a promising alternative anti-leishmanial agent, marred by solubility and bio-distribution limitations. Nowadays the use of solubilizing agents like Cremophor EL in pharmaceutical applications is limited as it induces severe anaphylactic hypersensitivity reactions, hyperlipidemia, abnormal lipoprotein patterns, aggregation of erythrocytes, and peripheral neuropathy in patients [38]. Therefore, biocompatible polymeric nanoparticles for AG was attempted with FDA approved biopolymer PLGA to overcome its biopharmaceutical problems. Both AG and polymer PLGA were proportionately soluble in volatile solvent chloroform which aided nanoparticle preparation by solvent evaporation process. The compound D-α-tocopheryl poly ethylene glycol 1000 succinate (TPGS) has been routinely used as a stabilizer and is a potent efflux pump inhibitor [39] and thereby inducing the efficacy of AGnps even against resistant leishmanial strains. Efflux pump (e.g., P-gp, MRP1 and BCRP) inhibition had been recognized as a strategy to overcome multidrug resistance and improves drug’s bioavailability. Vitamin E TPGS is known to modulate efflux pump activity. Competitive inhibition of substrate binding, alteration of membrane fluidity and inhibition of efflux pump ATPase activity had been proposed as possible mechanisms. TPGS inhibited substrate induced ATPase activity without inducing significant ATPase activity on its own [40]. Nanoparticles in a size range of around 200 nm are associated with increased phagocytosis [41] and subsequent engulfment by macrophages infected with the leishmanial parasite. Acidic phagolysosomal pH [42] favors the autocatalytic degradation of PLGA nanoparticles [43] leading to release of therapeutic burden and assures localized effect of antileishmanial agents.

SSG and PMM resistant cell lines AG83-R were 32 to 38 times more resistant than wild-type AG83, whereas field isolate GE1 strain was only 2 to 3 times more resistant (Table 2). In contrast, amphotericin B was equipotent to wild-type AG83, AG83-R and GE1 strains. Similarly, RI revealed that AG had equipotent activity on AG83-R and GE1 strains. Incorporation of AG in nanoparticles with TPGS and without TPGS did not significantly alter the RI values in AG83-R and GE1 strains. From these observations we may conclude that both free AG and nanoparticulated AG can overcome the drug resistance in axenic amastigotes. In cellular evaluation model (Table 3), amphotericin B showed similar RIs in AG83-R and GE1 strains, whereas SSG and PMM showed approximately 3 times less RIs in AG83-R compared to axenic amastigote model (Table 2). It appeared from this result that macrophage cells could actively concentrate the drug. Here, it was noteworthy that RIs of AG, AGnp with TPGS and AGnp without TPGS in axenic and cellular evaluation models were almost similar. If we consider the SI, amphotericin B revealed maximum selectivity among the others for wild-type AG83, AG83-R and GE1 strains. Preparation of AGnp with TPGS resulted in approximately 3.5 times greater SI than that of AGnp without TPGS for wild-type AG83, AG83-R and GE1 strains (Table 3). In the current study, we have successfully developed a PLGA-TPGS based nanocarrier for delivery of the hydrophobic plant bioactive AG. The AG-loaded PLGA-TPGS nanoparticles exhibited the following properties: bio-applicablity and bio-absorbability of PLGA-TPGS nanoparticles; high loading capacity; a controlled drug release profile; and markedly increased SI values for both wild-type and drug resistant strains of *Leishmania* parasites. Further studies, directed towards *in vivo* efficacy evaluations of AG nano-scale devices are perquisite for this newer antileishmanial to enter clinical stages.

## Conclusions

AG loaded PLGA (50∶50) nanoparticles stabilized by vitamin E TPGS were prepared for delivery into macrophage cells infested with sensitive and drug resistant amastigotes of *Leishmania* parasites. Antileishmanial activity as revealed from selectivity index in wild-type strain was found to be significant for AGnp with TPGS in about one-tenth of the dosage of the free AG and one-third of the dosage of the AGnp without TPGS. Cytotoxicity of AGnps with and without TPGS was significantly less than standard antileishmanial chemotherapeutics like amphotericin B, paromomycin or sodium stibogluconate. In summary, results from the study of one wild-type and three resistant *Leishmania* strains indicated that andrographolide was not the subject to the action of P-glycoproteins and MRP1. When andrographolide was incorporated in TPGS nanoparticles, selectivity of andrographolide increased eight-fold in resistant cells. This information strengthens the possibility of and enhances the impetus for developing andrographolide as a therapeutic agent for the treatment of leishmaniasis.
